# Autoimmune Hemolytic Anemia Secondary to COVID-19 Vaccine: A Case Report

**DOI:** 10.7759/cureus.46678

**Published:** 2023-10-08

**Authors:** Mitul H Chaudhary, Anil Kumar Yennam, Nagavenakata Lova Surya Vamshi Avinash Bojanki, Alyssa Nicole G Dela Cruz, Nirali K Chaudhary, Hitesh Kinha, Yoshita Rao Annepu

**Affiliations:** 1 Medicine, AMA School of Medicine, Makati, PHL; 2 Medicine, Emilio Aguinaldo College, Manila, PHL; 3 Medicine, Siddhartha Medical College, Vijayawada, IND; 4 Medicine, De La Salle Medical and Health Sciences Institute, Manila, PHL; 5 General Medicine, Dr. D. Y. Patil Medical College, Hospital & Research Centre, Pune, IND; 6 Medicine, Rangaraya Medical College, Kakinada, IND

**Keywords:** autoimmune haemolytic anemia, covishield, vaccine, acquired hemolysis, autoimmune

## Abstract

Autoimmune hemolytic anemia (AIHA) is an acquired hemolysis caused by one's immune system targeting red blood cell surface antigens, resulting in a shortening of the normal red-cell lifespan of 120 days. In this case report, we present an unusual case of a middle-aged woman with no known autoimmune diseases. After ruling out all other possible etiologies, she was later diagnosed with AIHA about two months after receiving her first dose of the Oxford-AstraZeneca COVID-19 vaccine (COVISHIELD). We discuss the possible underlying cause, the COVID-19 vaccine, for the precipitation of AIHA. The importance of identifying rare adverse events that could occur during mass vaccination is highlighted in this case. The patient was treated with oral steroids and received three blood transfusions. She was discharged after 21 days from the hospital and followed up after six months with no relapse.

## Introduction

The COVID-19 pandemic has been one of the greatest health crises in history. To limit the spread of the virus, multiple vaccines have been developed and approved. Along with the common side effects, these vaccines also include some rare adverse effects. New-onset autoimmune phenomena after COVID-19 vaccination has been recently reported. Autoimmune hemolytic anemia (AIHA) is an acquired hemolysis caused by the host's immune system acting against its red blood cells [1]. Molecular mimicry, the production of specific autoantibodies, and certain vaccine adjuvants seem to play a significant role in this autoimmune phenomenon [2]. Despite the numerical difference in the frequency of these events, we report a rare case of warm antibody AIHA secondary to the Oxford-AstraZeneca COVID-19 vaccine (COVISHIELD) manufactured by the Serum Institute of India.

## Case presentation

A 53-year-old female homemaker presented to the emergency department with complaints of generalized weakness, breathlessness on exertion, yellowish discoloration of the sclera for the past 10 days, and passing dark-colored urine for the past seven days. There was no history of drug or toxin exposure, acute or chronic illnesses, blood transfusions, comorbidities, or autoimmune diseases in the past. The patient had no history of passing blood in vomit or stool and no history of passing red-colored urine. She had received first dose of the Oxford-AstraZeneca COVID-19 vaccine (COVISHEILD) two months prior. She also has not received any other vaccines recently.

On examination, the patient was afebrile, with a heart rate of 116 beats per minute, a respiratory rate of 28 cycles per minute, a blood pressure of 100/80 mm Hg, and an oxygen saturation of 97% on room air. The patient was conscious and oriented to time, place, and person, with a Glasgow Coma Scale (GCS) score of 15/15. Other systemic examinations were unremarkable, with moderate pallor and mild icterus present.

Laboratory analysis (Table 1) showed a hemoglobin level of 4.8 g/dL, a total leucocyte count (TLC) of 7100 cells/dL, a platelet count of 261,000 cells/dL, and a hematocrit of 14.5%. Liver function tests revealed a total bilirubin level of 7.1 mg/dL, with an indirect fraction of 4.29 mg/dL and normal enzyme levels. The urine analysis was normal. The reticulocyte count was 6%, and the corrected reticulocyte count was 4%. Direct anti-globulin testing (DAT) showed 4+ IgG without C3, haptoglobin was <30 mg/dL, and lactate dehydrogenase (LDH) was 2566 U/L. A peripheral blood smear examination showed anisopoikilocytosis with hypochromia, a moderate number of spherocytes, and no sickle cells or target cells. A bone marrow study showed a hypercellular marrow with erythroid hyperplasia and a normal myeloid lineage. The ECG was suggestive of sinus tachycardia. Imaging studies, including chest X-ray and USG abdomen, were both normal, with no evidence of organomegaly.

**Table 1:** Laboratory parameters on admission TLC: total leucocyte count, MCV: mean corpuscular volume, RDW: red cell distribution width, ANA-IF: antinuclear antibody by immunofluorescent method, G6PD levels: glucose-6 phosphate dehydrogenase enzyme levels, DCT: direct Coomb test, C3: compliment protein 3, C4: compliment protein 4, ANCA: anti-neutrophil cytoplasmic antibodies, anti-SARS-CoV-2 IgG antibody: anti-severe acute respiratory syndrome coronavirus-2 IgG antibody, anti-CMV IgM/IgG antibodies: anti-cytomegalovirus IgM/IgG antibodies, anti-EBV IgM/IgG antibodies: anti Epstein Barr virus IgM/IgG antibodies

Test	Result	Reference range
Hemoglobin	4.8 g/dL	13.2-16.6 mg/dL
TLC	7100 cells/µl	4000-11000 cells/µl
MCV	85.9	80-100
Platelets	261,000 cells/µl	150 000-450 000 cells/µl
RDW	15.7%	12-14%
Hematocrit	14.5 %	45-55%
Reticulocyte count	6 %	
Haptoglobin	<30 mg/dL	40-200 mg/dL
Total bilirubin	7.10 mg/dL	0.1-1.2 mg/dL
Direct bilirubin	2.81 mg/dL	0-0.3 mg/dL
Indirect bilirubin	4.29 mg/dL	0.2-0.9 mg/dL
LDH	2566 U/L	85 to 227 U/L
Urea	71 mg/dL	17-49 mg/dl
Creatinin	1.01 mg/dL	0.6-1.2 mg/dl
CRP	2 mg/L	<2 mg/l (low cardiovascular risk)
PT	12.06 seconds	11-13.5 seconds
INR	1.1	0.8-1.1
Total protein	7.2 g/dL	6-8.3 g/dL
Albumin/globulin ratio	1.2	1.1-2.5
Folate	8 ng/mL	2.7-17 ng/mL
Vitamin B12	940 pg/mL	180-1000 pg/mL
Iron	174 µg/dL	40-190 µg/dL
Total iron binding capacity	249 µg/dL	150-450 µg/dL
Transferrin saturation	69.8%	11-50%
Ferritin	811 ng/mL	21.81 to 274.66 ng/mL
Uric acid	5.2 mg/dL	3.5-7.2 mg/dL
Calcium	8.8 mg/dL	8.5-10.5 mg/dL
HIV/HCV/HBsAg	Non-reactive	
G6PD levels	>20 units/gm of Hb	8-18.6 units/gm of Hb
DCT	Strongly positive (4+ IgG without C3)	
ANA IF	Negative	
Rheumatoid factor	Negative	
C3	Normal	
C4	Normal	
C-ANCA	Negative	
P-ANCA	Negative	
Anti-SARS-CoV-2 IgG antibody	Positive	
Anti-CMV IgM/IgG antibodies	Negative	
Ant-EBV IgM/IgG antibodies	Negative	
Blood culture	No Growth	
Urine culture	No Growth	
Urine routine/microscopy		
Color	Dark brown color	Pale yellow
Proteins	Trace	Absent
RBC	10-15 RBC/hpf	<5 RBC/hpf
Bile pigments and salts	Absent	Absent
Pus cells	Absent	Absent
Epithelial cells	Absent	Absent
Urine hemoglobin	Positive	Negative
Peripheral blood smear	Anisopoikilocytosis with hypochromia, a moderate number of spherocytes, no sickle cells, and no target cells	
Bone marrow aspiration and biopsy	Hypercellular marrow with erythroid hyperplasia, normal myeloid lineage	

The clinical presentation and laboratory parameters were consistent with hemolysis, leading to the diagnosis of AIHA. Further investigations were conducted to identify the underlying cause. Blood and urine cultures were negative. The serology for hepatitis C virus (HCV), human immunodeficiency virus (HIV), Epstein Barr virus (EBV), and cytomegalovirus (CMV) was negative. Rheumatoid factor and antinuclear antibody were also negative (Table 1).

After ruling out possible underlying etiologies for AIHA, such as drug, toxin, viral or bacterial infection, malignancies, and other autoimmune diseases, and given the recent correlation with the COVISHIELD vaccination with positive anti-SARS-CoV-2 IgG antibody titers, the possibility of COVID-19 vaccine-induced AIHA was considered. The patient was started on tab prednisone at 1 mg/kg/day after consultation with a hematologist and received three units of packed red blood cells (PRBCs). Hemoglobin and hematocrit began to normalize within two weeks of treatment, and there was no relapse thereafter.

## Discussion

AIHA following the COVID-19 vaccine is a rare manifestation of the disease. For COVID-19, more than 200 vaccines are being developed, including conventional inactivated vaccines, mRNA vaccines, protein subunit vaccines, and viral vector vaccines. Both COVAXIN (an inactivated vaccine) and COVISHEILD (a viral vector vaccine) are readily available in India [3]. In our patient, clinical and laboratory findings were consistent with hemolysis, leading to the diagnosis of AIHA. After excluding all possible underlying etiologies for AIHA and considering the recent correlation with the COVISHIELD vaccine, we assumed the possibility of AIHA following vaccination, which was later supported by the patient's response to oral steroids.

COVID-19 vaccine-induced AIHA is seldom reported, with only three case reports so far, all secondary to mRNA-based COVID-19 vaccines [4,5]. To the best of our knowledge, this is the first time that AIHA has been reported in an exclusive association with the Oxford-AstraZeneca COVID-19 vaccine (COVISHEILD). The direct causal relation between COVID-19 vaccination and AIHA is yet questionable. One theory proposes that it is an autoimmune response due to molecular memory of host antigens causing the polyclonal activation of T or B cells, which cross-react with red cell surface antigens [4,6]. Post-vaccination AIHA is manageable, and the advantages of immunization can outweigh these rare risks.

We propose that the delayed onset (two months after the first dose of the vaccine), moderate severity, and different immunohematology (strong IgG4+ without C3) suggest that the case reported here may involve a different immunologic process.

## Conclusions

AIHA is a very rare complication of COVID-19 vaccination due to a dysregulated immune response. Significantly less is known about COVID-19 vaccination's long-term sequelae. Further research on this subject and continued monitoring of post-vaccinated patients may yield a better understanding of the hematologic effects of these novel agents. We discussed a rare case of AIHA in association with the COVISHEILD vaccine in the hope of spreading knowledge about this probably underdiagnosed autoimmune association.

## References

[1] Hill A, Hill QA (2018). Autoimmune hemolytic anemia. Hematology Am Soc Hematol Educ Program.

[2] Chen Y, Xu Z, Wang P, Li XM, Shuai ZW, Ye DQ, Pan HF (2022). New-onset autoimmune phenomena post-COVID-19 vaccination. Immunology.

[3] Chakraborty C, Bhattacharya M, Dhama K (2023). SARS-CoV-2 vaccines, vaccine development technologies, and significant efforts in vaccine development during the pandemic: the lessons learned might help to fight against the next pandemic. Vaccines (Basel).

[4] Gaignard ME, Lieberherr S, Schoenenberger A, Benz R (2021). Autoimmune hematologic disorders in two patients after mRNA COVID-19 vaccine. Hemasphere.

[5] Murdych TM (2022). A case of severe autoimmune hemolytic anemia after a receipt of a first dose of SARS-CoV-2 vaccine. Int J Lab Hematol.

[6] David P, Shoenfeld Y (2020). ITP following vaccination. Int J Infect Dis.

